# Immunoexpression profile of hypoxia-inducible factor (HIF) targets in potentially malignant and malignant oral lesions: a pilot study^*^

**DOI:** 10.1590/1678-7757-2022-0461

**Published:** 2023-05-15

**Authors:** Shakiba GHOLAMI, Cintia CHAMORRO-PETRONACCI, Mario PÉREZ-SAYÁNS, José SUÁREZ PEÑARANDA, Adhemar LONGATTO-FILHO, Fátima BALTAZAR, Julieta AFONSO

**Affiliations:** 1 University of Minho School of Medicine Life and Health Sciences Research Institute Braga Portugal University of Minho, School of Medicine, Life and Health Sciences Research Institute (ICVS), Braga, Portugal.; 2 University of Minho Government Associate Laboratory Guimarães Portugal University of Minho, ICVS/3B’s – PT Government Associate Laboratory, Guimarães, Portugal.; 3 Universidad de Santiago de Compostela Facultad de Medicina y Odontología Unidad de Medicina Oral, Cirugía Oral e Implantología Santiago de Compostela España Universidad de Santiago de Compostela, Facultad de Medicina y Odontología, Unidad de Medicina Oral, Cirugía Oral e Implantología, Grupo MedOralRes, Santiago de Compostela, España.; 4 Universidad de Santiago de Compostela Instituto de Investigación Sanitaria de Santiago de Compostela Grupo ORALRES Santiago de Compostela España Universidad de Santiago de Compostela, Instituto de Investigación Sanitaria de Santiago de Compostela (IDIS), Grupo ORALRES, Santiago de Compostela, España.; 5 Universidade Estadual Paulista Faculdade de Medicina Laboratório de Investigação Médica São Paulo Brasil Universidade Estadual Paulista, Faculdade de Medicina, Laboratório de Investigação Médica (LIM 14), São Paulo, Brasil.; 6 Hospital do Câncer de Barretos Centro de Pesquisa em Oncologia Molecular São Paulo Brasil Hospital do Câncer de Barretos (Hospital de Amor), Centro de Pesquisa em Oncologia Molecular, São Paulo, Brasil.

**Keywords:** Head and neck cancer, Lactate transport, Monocarboxylate transporters, Aerobic glycolysis, Warburg effect

## Abstract

Oral potentially malignant disorders (OPMD) are associated with an increased risk of oral squamous cell carcinoma (OSCC). OSCC has an aggressive profile and is the most prevalent among different head and neck malignancies. Most OSCC patients are diagnosed with advanced stage tumors and have a poor prognosis. Cancer cells are able to reprogram their metabolism, even in the presence of oxygen, enhancing the conversion of glucose to lactate via the glycolytic pathway, a phenomenon mainly regulated by hypoxia-inducible factor (HIF) signaling. Thus, several glycometabolism-related biomarkers are upregulated.

**Objectives:**

This study aimed to evaluate the immunoexpression of the HIF targets GLUT1, GLUT3, HK2, PFKL, PKM2, pPDH, LDHA, MCT4, and CAIX in OPMD and OSCC samples, in order to identify potential correlations between biomarkers’ immunoexpression, clinicopathological features, and prognostic parameters.

**Methodology:**

OSCC and OPMD samples from 21 and 34 patients (respectively) were retrospectively collected and stained for the different biomarkers by immunohistochemistry.

**Results:**

CAIX and MCT4 expressions were significantly higher in OSCC samples when compared with OPMD samples, while the rest were also expressed by OPMD. GLUT3 and PKM2 alone, and the concomitant expression of more than four glycometabolism-related biomarkers were significantly correlated with the presence of dysplasia in OPMD. When considering OSCC cases, a trend toward increased expression of biomarkers and poor clinicopathological features was observed, and the differences regarding HK2, PFKL, LDHA and MCT4 expression were significant. Moreover, HK2 and CAIX were correlated with low survival rates. GLUT1 and GLUT3 were significantly associated with poor outcome when their expression was observed in the hypoxic region of malignant lesions.

**Conclusion:**

OPMD and OSCC cells overexpress glycolysis-related proteins, which is associated with aggressive features and poor patient outcome. Further research is needed to deeply understand the glycolic phenotype in the process of oral carcinogenesis.

## Introduction

Oral cancer (OC), which includes lip and oral cavity malignancies, is the most prevalent among different subtypes of head and neck cancers (HNC). The incidence rate is progressively increasing: 377,713 new cases and 177,757 OC-related deaths were estimated worldwide in 2020. OC occurs mainly in regions of South and Central Asia and Melanesia, with a high prevalence in men.^1^ In terms of etiology, both hereditary – family history of cancer and personal defective immune system – and non-hereditary factors – tobacco, alcohol, and HPV virus (high-risk HPV) – play an important role in oral carcinogenesis.^2^ Oral potentially malignant disorders (OPMD), such as oral lichen planus (OLP) and leukoplakia, are also associated with an increased risk of OC, especially lesions with high-grade dysplasia. Most OPMD are asymptomatic in the early stages of their evolution, which makes knowledge of their clinical aspects essential for a timely diagnosis and surveillance.^3^ Oral squamous cell carcinoma (OSCC) is the predominant histological type of OC. The successful treatment strategy for OSCC patients at an early stage is surgery followed by radiotherapy. In advanced stages, surgery is combined with cisplatin-based chemotherapy, which increases survival rates. Despite advancements in the treatment of OSCC, this malignancy is not usually detected in the early stages, and more than half of patients succumb to the disease within five years of diagnosis. Moreover, conventional treatments have significant medical costs and associated comorbidities.^2^ Therefore, it is necessary to search for new diagnostic and prognostic biomarkers that can improve patients’ outcome and quality of life.

Cancer cells, regardless of oxygen availability, use much higher glucose levels than their non-cancerous counterparts, with further fermentation of pyruvate to lactate rather than oxidation in the mitochondria. This phenomenon called the Warburg effect upregulates the expression of glycolytic enzymes and other glycometabolism-related proteins.^4^ In OSCC, similarly to other types of cancer, the activated HIF-1 (hypoxia-inducible factor 1) signaling pathway seems to be the main regulator of the Warburg phenotype.^5^ In this pathway, HIF-1α increases the expression of glucose transporters (GLUT), namely GLUT1 and GLUT3, which results in increased glucose uptake. During glucose metabolism, HIF-1α also promotes the conversion of glucose to pyruvate by increasing the expression of glycolytic enzymes such as hexokinase 2 (HK2; which catalyzes the conversion of glucose to glucose-6-phosphate II), phosphofructokinase (PFK; which accelerates the conversion of fructose-6-phosphate to fructose-1,6-bisphosphate, mainly the L type), and pyruvate kinase isoform M2 (PKM2; which catalyzes the conversion of phosphoenolpyruvate to pyruvate).^6^ Electron flux through oxidative phosphorylation is inhibited by pyruvate dehydrogenase kinase (PDK), a negative regulator of pyruvate dehydrogenase (PDH; which catalyzes the conversion of pyruvate to acetyl-CoA), and lactate dehydrogenase A (LDHA; which catalyzes the conversion of pyruvate to lactate), both under HIF-1 signaling.^7^ Lactate is forced out of the cell by monocarboxylate transporters (MCT), of which MCT4, the preferred lactate extruder in highly glycolytic tissues, is upregulated by HIF-1α.^8^ Under HIF-1α targeting, carbonic anhydrase IX (CAIX) catalyzes the reversible hydration of CO_2_, playing an important role in the regulation of cellular pH and, consequently, microenvironmental acidity.^9^ Thus, these proteins, by being involved in the glycolytic metabolism under direct HIF-1α targeting, are associated with the development of an acidic tumor microenvironment (TME), and seem to be implicated in the progression, dissemination, resistance to treatment, and poor prognosis of OSCC.^10,11^ Although these biomarkers have been investigated in the context of OSCC, comprehensive studies on their expression pattern from premalignancy to malignancy are scarce. These studies can offer valuable insights into the process of oral carcinogenesis and the progression of OC. Thus, this study aimed to evaluate the immunoexpression of the HIF targets GLUT1, GLUT3, HK2, PFKL, PKM2, phospho-PDH (pPDH), LDHA, MCT4, and CAIX in OPMD and OSCC lesions by immunohistochemistry, further characterizing their effect on the survival and clinicopathological parameters of patients. This was intended to be a pilot study involving a small but representative number of patients in order to select the most informative glycolysis-related biomarker(s) regarding clinical and prognostic associations, which should be further explored in future clinical and preclinical studies.

## Methodology

### Individuals and tissue samples

This retrospective study was approved by the Galician Autonomous Committee of Ethics (references 2013/541 and 2019/271). The experiments were conducted with the understanding and written consent of each participant and in line with the principles of the Declaration of Helsinki. In order to perform a pilot study, 55 formalin-fixed paraffin-embedded oral cavity lesions (34 OPMD and 21 OSSC cases) were randomly obtained from the Department of Oral Medicine, Oral Surgery and Implantology, University Hospital Complex of Santiago de Compostela, from patients admitted to the institution from October 1997 to February 2020 and from January 1992 to June 2019, respectively. Clinicopathological and/or follow-up information was extracted from the patients’ medical records. Only cases diagnosed with OPMD and OSSC lesions were collected for this study, according to the following exclusion criteria: diagnosis of OSCC with variant histology (spindle cell or sarcomatoid squamous cell carcinoma; pseudoglandular cell, basaloid cell, and small cell neuroendocrine carcinoma); follow-up time shorter than one month; and/or inadequate tissue samples for histological evaluation. Regarding OPMD patients, their mean age was 59 years (range 29–85); 18 (52.9%) were men and 16 (47.1%) were women. The mean age of OSCC patients was 67 years (range 50–95); 11 (52.4%) were men and 10 (47.6%) were women. OPMD and OSCC patients were classified according to the World Health Organization (WHO)^12^ and the latest version of the American Joint Committee on Cancer (AJCC) staging system,^13^ respectively. Clinical data collected from the records of OPMD patients included age, sex, lesion site, lesion size, tobacco and alcohol use habits, type and clinical presentation of the lesion, type of biopsy, type of treatment, and follow-up. Regarding OSCC patients, clinicopathological parameters included age, sex, tumor site, tumor size, tobacco and alcohol use habits, TNM stage (four categories), tumor grade, and follow-up data (disease recurrence and/or death). All data are shown in Table 1 and 2. For OSCC patients, the follow-up period was calculated from the date of admission to the last follow-up visit, and ranged from one to 28 months, with a mean and median follow-up of 14.6 and 16.7 months, respectively. Disease-free survival (DFS) was defined as the time from first treatment to recurrence; recurrence occurred in ten patients (47.6%). Overall survival (OS) was defined as the time from the first treatment to the last follow-up assessment or patient death; this occurred in eleven patients (52.4%).

**Table 1:** Overview of clinicopathological data in patients with oral potentially malignant disorders

Age	<59	17
	>59	17
Sex	Men	18
	Women	16
Biopsy site	Buccal mucosa	15
	Soft palate	3
	Hard palate	1
	Tongue	11
	Floor of the mouth	1
	Alveolar crest	1
	Keratinized gingiva	2
Lesion size	<0.8 cm	15
	>0.8 cm	19
Smoking habits	Non-smoker	15
	Former smoker	13
	Smoker	6
Alcohol use	No	21
	Yes	13
Type of lesion	White oral lichen planus	12
	OL without dysplasia	11
	OL with low grade dysplasia	8
	OL with high grade dysplasia	3
Clinical presentation	White oral lichen planus	12
	Homogeneous OL	13
	Verrucous NH OL	6
	Nodular NH OL	2
	Erythroleukoplakia	1
Type of biopsy	Incisional	29
	Excisional	5
Treatment	Follow-up	18
	Corticosteroids	4
	Full surgery	4
	CO^2^ laser vaporization	8
Follow-up	Resolution	5
	Stable disease	24
	Recurrence	3
	Progression to OSCC	2

OL: oral leukoplakia; NH: non-homogenous; OSCC: oral squamous cell carcinoma.

**Table 2:** Overview of clinicopathological data in patients with oral squamous cell carcinoma

Age	<67	10
	>67	11
Sex	Men	11
	Women	10
Biopsy site	Buccal mucosa	5
	Soft palate	1
	Hard palate	1
	Tongue	5
	Maxilla	3
	Floor of the mouth	3
	Alveolar crest	3
Tumor size	<2.5 cm	10
	>2.5 cm	11
Smoking habits	Non-smoker	11
	Former smoker	4
	Smoker	6
Alcohol use	No	14
	Yes	6
	Former user	1
TNM stage	I	5
	II	5
	III	1
	IV	10
Grade	Well-differentiated	10
	Moderately differentiated	10
	Poorly differentiated	1
Recurrence	No	11
	Yes	10
Death	No	10
	Yes, from causes other than OSSC	3
	Yes, from OSCC	8

TNM: (T) primary tumor, (N) regional lymph nodes, and (M) distant metastasis; OSCC: oral squamous cell carcinoma.

### Immunohistochemistry and Evaluation of Immunohistochemistry Results

Immunohistochemistry protocols were performed on 4 μm-thick representative sections of the collected samples. The Thermo Scientific™ Lab Vision™ UltraVision™ Large Volume Detection System: anti-Polyvalent, Horseradish peroxidase (HRP) (Thermo Fisher Scientific, Waltham, MA, USA), based on the streptavidin-biotin peroxidase principle, was used for the detection of PKM2, PFKL, GLUT1, and HK2. The Thermo Scientific™ Lab Vision™ UltraVision™ ONE Detection System: HRP Polymer (Thermo Fisher Scientific, Waltham, MA, USA), based on the polymeric method principle, was used for the detection of LDHA, pPDH, CAIX, MCT4 and GLUT3. Color development was evaluated by the Thermo Scientific™ 3,3′-diaminobenzidine colorimetric substrate (Thermo Fisher Scientific, Waltham, MA, USA). All procedures were performed according to the manufacturer’s instructions. Positive and negative controls were included. Details are presented in Table S1.

**Figure 1:**
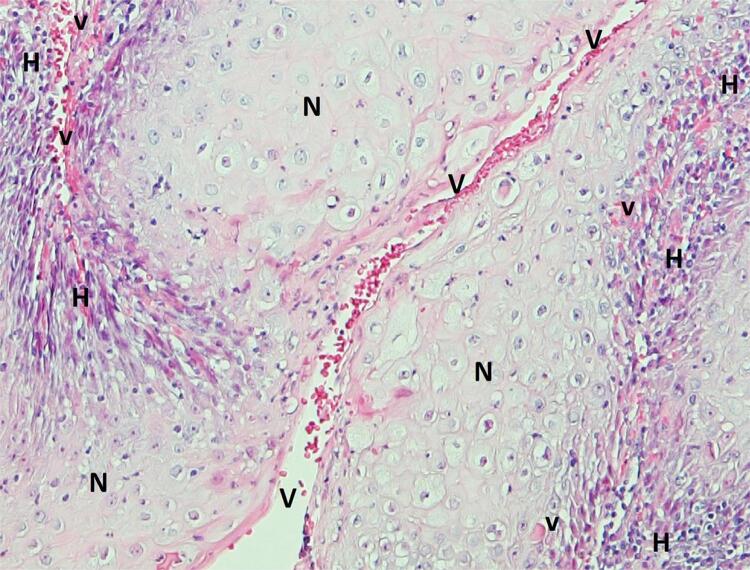
Representative images of a section of oral squamous cell carcinoma stained with haematoxylin and eosin, showing normoxic regions (N) with non-distorted blood microvessels (V) and hypoxic regions (H) with distorted and packaged vessel structures with haemorrhagic spots (v)

For the evaluation of immunohistochemistry results, two independent pathologists semi-quantitatively scored stained tissues—the discordant cases were re-evaluated and classified by consensus. The grading system used (Figure S1) considered the extension of expression, showing the concentration of target proteins, which were grouped into 0 (0%), 1 (0–5%), 2 (5–50%), and 3 (>50%) of immunoreactive cells. Staining intensity was graded as 0 (negative), 1 (weak), 2 (moderate), or 3 (strong). Extension and intensity scores were summed to obtain the final score and clustered as negative (score 0–2 or 0–3) and positive (score 3–6 or 4–6), depending on the biomarker. The final scores that provided the most informative results regarding clinicopathological and prognostic implications for each studied biomarker were considered. Protein localization (cytoplasm, nucleus, and/or cell membrane) was also assessed. OPMD were uniformly assessed for protein expression and OSCC sections (cluster of malignant cells/tumor mass) were evaluated semi-quantitatively in areas near (normoxic region) and far (hypoxic region) from non-distorted blood microvessels (Figure 1). Only groups of endothelial cells organized around a visible lumen separated from other microvessels or stromal components were considered non-distorted blood microvessels, since abnormal structures result in reduced transport of oxygen and nutrients that, associated with increased cancer cell proliferation and altered metabolism, lead to the development of hypoxia. Immunohistochemistry with CD31, a specific marker for blood endothelial cells, was used in doubtful cases, in which no red blood cells were observed in the lumens and the vascular structures had a distorted and packaged appearance.^14^

### Statistical analysis

Data analyses were performed using the Statistical Package for Social Sciences (SPSS version 25; IBM Company, Armonk, NY, USA). Correlations of biomarker immunoexpression in OPMD and OSCC samples and associations with clinicopathological parameters were assessed using Pearson’s chi-square (χ^2^) and Fisher’s exact tests. DFS and OS rates were evaluated by the Kaplan-Meier method and the analysis was compared using the log-rank or Breslow tests. A p-value of <0.05 was considered significant.

## Results

### Prognostic Significance of Clinicopathological Parameters in OPMD and OSCC

All OPMD patients were alive at their last clinical assessment, as expected. In total, 91.7% of patients with white OLP had stable disease, while the two patients with tumor progression had leukoplakia with dysplasia (p<0.008; data not shown). Regarding cancer cases, seven of the eight patients (87.5%) who died from OSCC had previously developed a recurrent disease (p=0.009; data not shown). Detailed results on the association between clinicopathological data and survival rates of OSCC patients are presented in Tables S2 and S3.

### Immunoexpression of Glycometabolism-Related Biomarkers in OPMD and OSCC

As previously mentioned, we considered the scores that allowed us to obtain the most informative results as the final staining scores for each biomarker. Thus, positivity was considered as >3 for GLUT1, GLUT3, PFKL, pPDH, LDHA, MCT4, and CAIX, and >4 for PKM2 and HK2. Significant differences were obtained when comparing the immunoexpression frequencies of CAIX (p<0.001) and MCT4 (p=0.011) in OPMD (uniform assessment of protein expression) and OSCC lesions (assessment of protein expression in normoxic regions), with a higher percentage of positive cases for these biomarkers in OSCC samples. An inverse correlation was observed when GLUT3 (p=0.033) and PFKL (p=0.023) expression frequencies were analyzed. Detailed information on the frequencies of biomarker immunoexpression is shown in Table 3.

**Table 3:** Immunoexpression frequencies of GLUT1, GLUT3, HK2, PFKL, PKM2, pPDH, LDHA, MCT4, and CAIX in oral potentially malignant disorders and cases of oral squamous cell carcinoma

	Oral potentially malignant disorders		Oral squamous cell carcinoma		
Biomarker	n	Positive (%)	n	Positive (%)	p
GLUT1	34	34 (100%)	19	18 (94.7%)	.358
GLUT3	34	14 (41.2%)	17	2 (11.8%)	**.033**
HK2	34	14 (41.2%)	19	5 (26.3%)	.279
PFKL	34	16 (47.1%)	19	3 (15.8%)	**.023**
PKM2	34	8 (23.5%)	18	3 (16.7%)	.564
pPDH	33	23 (69.7%)	19	8 (42.1%)	.051
LDHA	34	9 (26.5%)	17	4 (23.5%)	.820
MCT4	34	0 (0.0%)	18	4 (22.2%)	**.011**
CAIX	34	2 (5.9%)	19	15 (78.9%)	**<0.001**

p-Values from Pearson's chi-square or Fisher’s exact tests, for the comparison between oral potentially malignant disorders and oral squamous cell carcinoma lesions. The p-values <0.05 are in bold.

**Figure 2:**
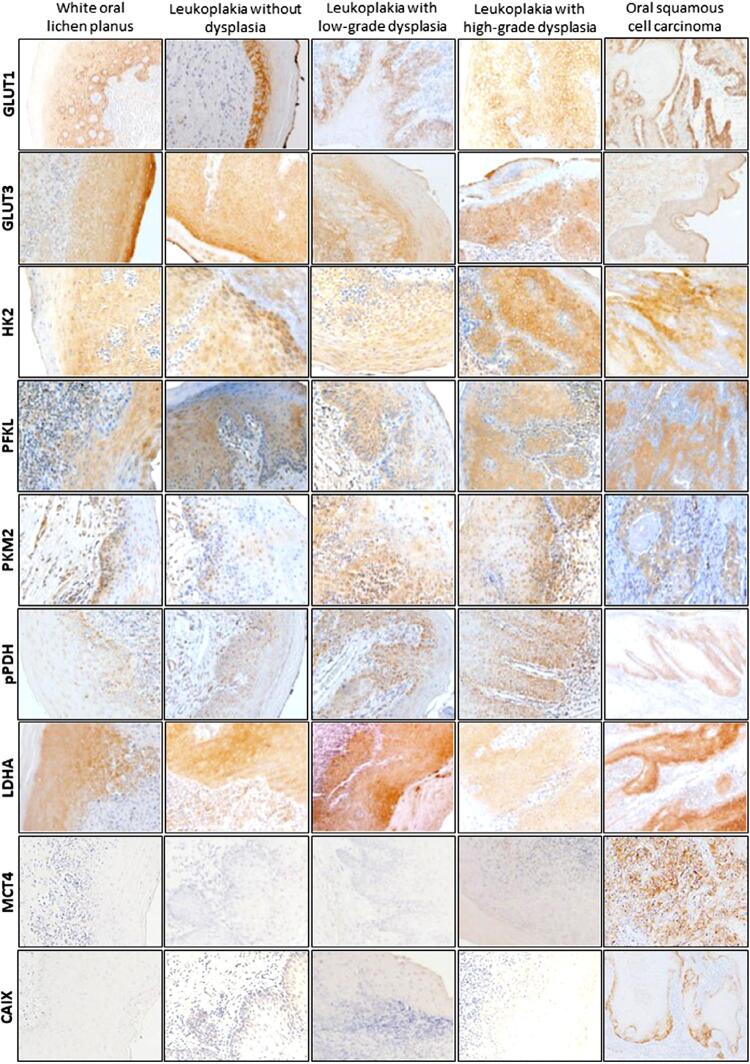
Representative images of the immunohistochemical staining reactions of GLUT1, GLUT3, HK2, PFKL, PKM2, pPDH, LDHA, MCT4, and CAIX in oral potentially malignant disorders and cases of oral squamous cell carcinoma

The expression of HK2, PFKL, PKM2, pPDH and LDHA was observed in the cytoplasm of stained cells, while GLUT1 and CAIX expression was mostly membranous. MCT4 and GLUT3 were found in both the membrane and the cytoplasm. Regarding PKM2 expression, occasional nuclear staining was observed in few OPMD and OSCC cases. Positive immunoexpression was found in endothelial cells with PKM2 and pPDH biomarkers, but only in OPMD. Representative images of the immunohistochemical reactions in the different types of lesions are presented in Figure 2 (uncropped images in Supplementary Data, Figures S2 to S10).

### Clinicopathological and Prognostic Significance of Glycometabolism-Related Biomarkers in OPMD and OSCC

The associations between the immunoexpression frequencies of glycometabolism-related biomarkers and the clinicopathological and prognosis parameters of the patients were assessed in this study. Significant clinicopathological associations are presented in Table 4.

**Table 4:** Significant clinicopathological associations resulting from positive [or negative (-), when indicated] immunoexpression of GLUT1, GLUT3, HK2, PFKL, PKM2, pPDH, LDHA, MCT4, and CAIX in oral potentially malignant disorders and cases of oral squamous cell carcinoma (normoxic and hypoxic regions)

	Clinicopathological associations		
Biomarker	OPMD	OSCC (normoxic regions)	OSCC (hypoxic regions)
GLUT1			
GLUT3	Leukoplakia with high-grade dysplasia (p=0.039)		
HK2	Women (p=0.005)	Age >67 (p=0.033) Tumor size >2.5 cm (p=0.033)	
PFKL			Tumor size >2.5 cm (p=0.001)
PKM2	Leukoplakia with high-grade dysplasia (p=0.044)		
pPDH	(-) Non-smokers (p=0.046)		(-) Tumor size >2.5 cm (p=0.033)
LDHA		Loss of differentiation (p=0.047)	
MCT4			Loss of differentiation (p=0.044)
CAIX		Non-smokers (p=0.040)	

The clinicopathological parameters under statistical analysis include age, sex, lesion size, smoking habits (for both OPMD and OSCC cases), type of lesion (for OPMD), TNM stage, and grade (for OSCC cases). The p-values <0.05 are in bold. OPMD: oral potentially malignant disorders; OSCC: oral squamous cell carcinoma.

Regarding OPMD, all patients with leukoplakia with high-grade dysplasia (3/3) expressed GLUT3, while only 16.7% of white OLP lesions were positive for this biomarker (p=0.039). The same was observed regarding PKM2 positivity (p=0.044). HK2 positivity was mostly observed in women (p=0.005). While pPDH expression was observed in 50% of non-smokers (7/14), 92.3% (12/13) of former smokers and 66.7% (4/6) of smokers had pPDH positivity (p=0.046). Regarding OSCC cases (normoxic regions), a significant association was observed between HK2 immunoexpression and advanced age (p=0.033). Moreover, all lesions larger than 2.5 cm were positive for HK2 (p=0.033). The vast majority of well-differentiated and moderately differentiated lesions were negative for PFKL and PKM2 expression, while the only poorly differentiated tumor existing in this study was positive. The same trend was observed with increasing TNM stage, although the differences were not significant. LDHA expression was also significantly associated with loss of differentiation (p=0.047) and a non-significant trend was observed on LDHA positivity, being higher in larger tumors. MCT4 expression increased with increasing TNM stage and loss of differentiation (not significant differences). All tumors from non-smoker patients (9/9) expressed CAIX (*p*=0.040). On the other hand, a trend toward CAIX positivity was observed with increasing TNM stage. Detailed results are shown in Supplementary Data (Tables S4 and S5).

**Figure 3:**
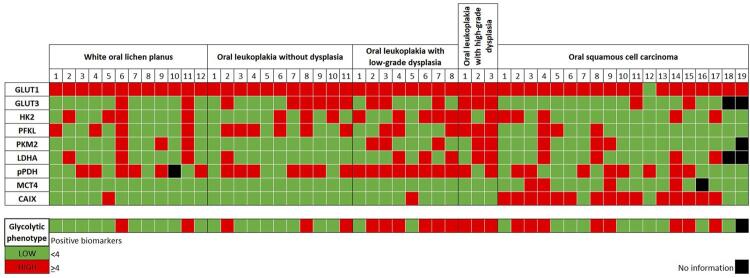
Detailed heat map of the immunoexpression results of GLUT1, GLUT3, HK2, PFKL, PKM2, pPDH, LDHA, MCT4, and CAIX in oral potentially malignant disorders and cases of oral squamous cell carcinoma (normoxic regions) for the development of a glycolytic phenotype

To perform an integrative analysis of the immunoexpression results, we developed a glycolytic phenotype (GP) model, in which we considered the cases with less than four positive biomarkers as low GP and the cases with four or more positive biomarkers as high GP. A detailed heat map is presented in Figure 3. Regarding OPMD, we grouped the cases based on the type of lesion. Patients with OLP (83.3%; 10/12) and leukoplakia without dysplasia (72.7%; 8/11) were predominantly in the low GP group, while patients with leukoplakia with low- (75%; 6/8) and high-grade dysplasia (100%; 3/3) were mostly in the high GP group. The differences were significant when comparing the four groups (p=0.007) and significance increased when comparing non-dysplastic (78.3%; 18/23 in the low GP) and dysplastic (81.9%; 9/11 in the high GP) OPMD (p=0.007). No statistically significant difference was observed when considering the remaining clinicopathological parameters. Regarding OSCC patients, there was a balanced distribution of the cases between the low (55.6%; 10/18) and high (44,4%; 8/18) GP groups, and no difference was observed when comparing these cases with OPMD cases. Well-differentiated OSCC lesions (87.5%; 7/8; p=0.031) were predominantly in the low GP group. An OSCC patient in whom only one positive immunoreaction was obtained (patient #12) had a small (<2.5 cm) well-differentiated stage I tumor.

**Figure 4:**
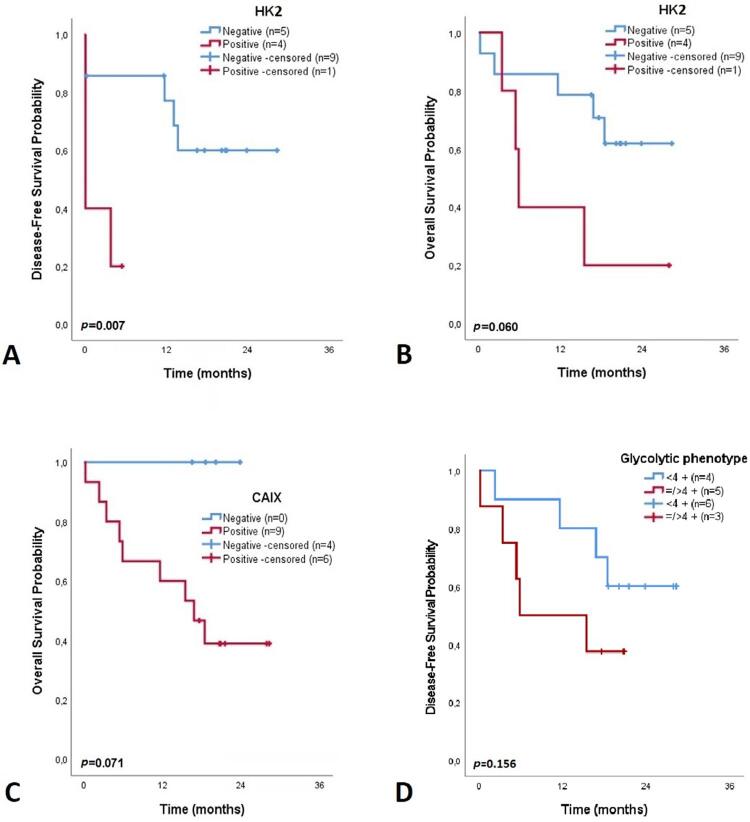
Kaplan-Meier curve showing 3-year disease-free survival, based on the immunoexpression status of HK2 (A), and 3-year overall survival, based on the immunoexpression status of HK2 (B) and CAIX (C) (normoxic regions) and the glycolytic phenotype (according to the number of positive biomarkers), of patients with oral squamous cell carcinoma

Kaplan-Meier survival analysis of OSCC patients regarding biomarker immunoexpression revealed that high HK2 expression was significantly correlated with worse DFS (p=0.007; Figure 4A) and nearly significantly associated with worse OS (p=0.060; Figure 4B). Moreover, a clear separation regarding OS rates was obtained between the negative and positive CAIX groups (p=0.071; Figure 4C) and between the low and high GP groups (p=0.156; Figure 4D), although the differences were not significant. Detailed results are presented in Tables S6 and S7.

### Immunoexpression of Glycometabolism-Related Biomarkers in Normoxic versus Hypoxic Regions of Malignant Lesions

**Figure 5:**
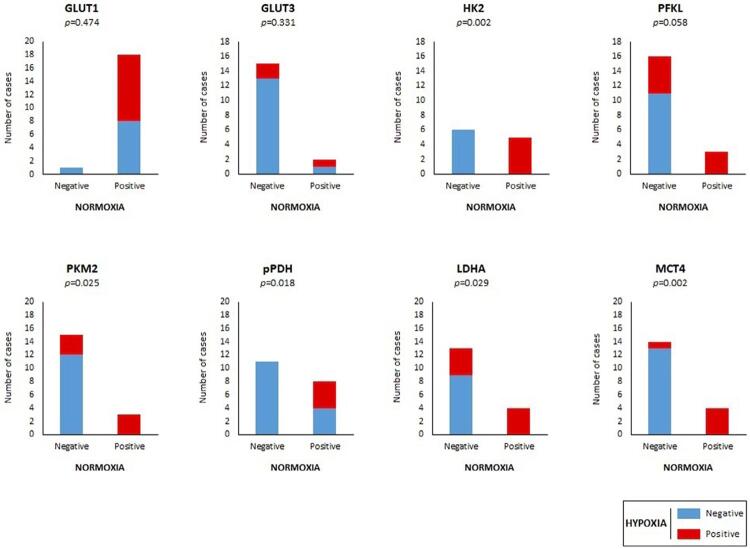
Immunoexpression of GLUT1, GLUT3, HK2, PFKL, PKM2, pPDH, LDHA, and MCT4 in normoxic versus hypoxic regions of cases of oral squamous cell carcinoma

A detailed analysis of tissue sections of OSCC lesions was performed regarding the expression of biomarkers in normoxic versus hypoxic regions. It was not possible to distinguish between normoxic and hypoxic regions regarding CAIX immunoexpression. There was a significant concordance in the absence or presence of the immunoexpression of HK2, PFKL, PKM2, pPDH, LDHA, and MCT4 in both normoxic and hypoxic regions. In opposition, GLUT1 and GLUT3 expression in the two regions was discordant. Results are presented in Figure 5.

### Clinicopathological and Prognostic Significance of Glycometabolism-Related Biomarkers in Hypoxic Regions of Malignant Lesions

**Figure 6:**
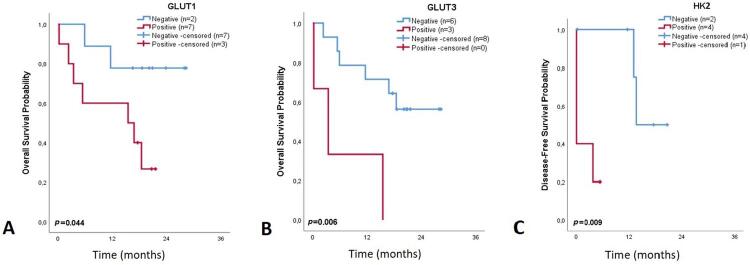
Kaplan-Meier curve showing 3-year overall survival, based on the immunoexpression status of GLUT1 (A) and GLUT3 (B), and 3-year disease-free survival, based on the immunoexpression status of HK2 (C) (hypoxic regions), of patients with oral squamous cell carcinoma

The associations between clinicopathological parameters and the immunoexpression frequencies of biomarkers in the hypoxic region of OSCC cases were assessed. A summary of the main results is shown in Table 4. Once concordance was obtained regarding the immunoexpression of HK2, PFKL, PKM2, pPDH, LDHA, and MCT4 in both normoxic and hypoxic regions, the associations with clinicopathological parameters were generally similar in the two conditions, as expected. Thus, HK2 (p=0.061) and PFKL (p=0.001) positivity was mostly observed in larger tumors. Moreover, nearly significant associations were obtained between HK2 expression and increasing age (p=0.061) and between PFKL expression and increasing TNM stage (p=0.074). All tissue sections from tumors larger than 2.5 cm were negative for pPDH expression (p=0.033). MCT4 expression was mostly observed in tumors with higher TNM stage (p=0.083) and grade of differentiation (p=0.044), and the same trend was also observed for LDHA positivity and TNM stage (p=0.072). The detailed analysis is shown in Table S8 (Supplementary Data). GLUT1 and GLUT3 immunoexpression did not associate with the clinicopathological parameters, but significant associations were observed between positivity of these biomarkers in the hypoxic region of tumors and a low overall survival rate (p=0.044 and p=0.006, respectively; Figures 6A and 6B). HK2 expression was significantly associated with poor disease-free survival (p=0.009; Figure 6C). Detailed results are presented in Tables S9 and S10 (Supplementary Data).

## Discussion

In this study, we sought to evaluate the expression of glycometabolism-related biomarkers in the process of oral carcinogenesis, specifically from OPMD to malignancy. The expression pattern of some glycometabolism-related proteins in oral lesions has been evaluated,^10^ although those studies have mainly focused on OSCC rather than potentially malignant conditions. Thus, we conducted a pilot study with 34 OPMD and 21 OSCC cases. The low number of cases within each group does not allow simple conclusions to be drawn regarding the correlation between clinicopathological parameters and prognosis. Even so, the coexistence of dysplasia in oral leukoplakia lesions was associated with progression to OSCC and cancer recurrence was associated with death from OSCC.

Immunohistochemistry was performed on tissue sections to evaluate the expression pattern of GLUT1, GLUT3, HK2, PFKL, PKM2, pPDH, LDHA, MCT4, and CAIX. The expression of the biomarkers was in accordance with their cellular location and function. A hyperglycolytic metabolism, supported by the overexpression of MCT4 and CAIX, in OSCC over OPMD was observed in our study, in line with other studies.^15^ An inverse correlation was found for GLUT3 and PFKL, while no differences were observed for the remaining biomarkers. Regarding the discrimination between hypoxic and normoxic regions, a significant immunoexpression concordance was obtained for most biomarkers, except for GLUT1 and GLUT3. As previously mentioned, the activated HIF-1 signaling pathway seems to be the main regulator of hyperglycolytic metabolism in OSCC,^5^ but such a phenotype may also be activated by stimuli other than hypoxia. In fact, HIF-1α stabilization may occur in a hypoxia-independent manner, and multiple transcriptional regulators, epigenetic events, or trafficking modulators are also capable of triggering metabolic reprogramming.^16^

The role of glucose transporters – especially GLUT1 and GLUT3 – in the pathophysiology of OSCC has been systematically reviewed, and these proteins are promising as diagnosis and prognosis biomarkers.^17^ GLUT1 is essential in mediating glycolytic metabolism, favoring cancer cell proliferation and survival.^16^ In OSCC, a significant correlation was found between GLUT1 expression and increased glucose uptake, proliferation, degree of dysplasia, increased depth of invasion, loss of differentiation, and distant metastasis.^18^ Moreover, this biomarker seemed to play a central role in the malignant transformation of oral epithelial dysplasia^18^ and leukoplakia,^19^ since its expression increased from premalignant to malignant stages, which was not observed in our study. In fact, in our study, most malignant cases, as well as OPMD samples, overexpressed GLUT1, suggesting an essential role of this biomarker since premalignancy. When its expression was observed in the hypoxic region of the cancer samples, it was significantly associated with lower overall survival. GLUT1 has been proposed by several authors as an endogenous marker for hypoxia in solid malignancies, including OC.^20^ In the central parts of lesions with inadequate glucose supply due to increased oxygen diffusion distance, hypoxia-stimulated GLUT1 expression increases to compensate for this condition, causing areas of squamous differentiation and/or keratinization in oral carcinomas.^18^

Similarly to GLUT1, GLUT3 mediates glucose uptake and its upregulation has also been reported in numerous types of cancer.^16^ In the study by Zhou, et al.^21^ (2008), GLUT3 gene expression in HNC was significantly higher when compared with non-malignant cases, and this was correlated with the occurrence of lymph node metastasis. In another study, GLUT3 expression was associated with the clinical stage of OSCC and low DFS among patients.^20^ In this study, GLUT3 had a restricted expression in OPMD and malignant lesions. Even so, and similarly to the results regarding GLUT1, although no major involvement of GLUT3 was evident in oral carcinogenesis and no association was found among clinical data, GLUT3 expression in the hypoxic region of cancer sections was significantly associated with a poor overall outcome. It is known that chronic hypoxia fluctuates with stages of normoxia recovery due to neovascularization and metastasis, which leads to increased metabolic activity and aggressiveness of cancer cells.^22^ This shows the importance of evaluating biomarker expression in a heterogeneous approach in order to assess the clinical and/or prognostic value, as well as the functional aspects of the metabolic heterogeneity intrinsic to the different regions of the tumor mass.

HK2 is the first rate-limiting enzyme in the glycolysis pathway, and is upregulated in multiple types of cancer.^23^ HK2 expression in OC has been reported,^24^ but we found no study on OPMD. Grimm, et al.^24^ (2014) showed the key role of HK2 in oral carcinogenesis, as its expression increased from normal mucosa to simple hyperplasia, squamous intraepithelial neoplasia, and OSCC tissues, where it correlated with poor survival of OSCC patients. In our study, HK2 expression was lower in OSCC samples than in OPMD samples. Interestingly, for patients with OPMD, HK2 expression was mostly seen in women. HK2 was the most informative biomarker for OSCC patients and our results showed a significant association between HK2 positivity and advanced age, large tumors, and poor prognosis.

PFKL is an important enzyme that controls the glycolytic flux and the only phosphofructokinase 1 isoform whose expression is directly affected by HIF-1α.^25^ To the best of our knowledge, no studies on PFKL expression in OPMD or OSCC have been reported. In esophageal cancer, high PFKL expression was associated with advanced stage tumors and poor patient survival.^26^ In our study, and similarly to HK2 expression, PFKL expression was higher in OPMD than in OSCC lesions. The few (three cases) cancer sections positive for PFKL were large advanced-stage high-grade tumors.

The glycolytic protein PKM2 is another HIF-1α target that favors cancer cell survival and invasion by aerobic metabolism.^27^ PKM2 expression has been reported to be significantly associated with OC progression and poor prognosis in OSCC patients, and also enhances VEGF-A expression (a direct target of HIF-1α with a major role in tumor angiogenesis).^28^ In our study, PMK2 expression in both OPMD and OSCC sections was low, but a higher expression was observed in OPMD with concomitant high-grade dysplasia, as well as in advanced-stage high-graded OSCC samples.

The PDH enzyme is responsible for converting pyruvate to acetyl-CoA, which then enters in the TCA cycle to form citrate and subsequently start oxidative phosphorylation (OXPHOS). This enzyme is a direct HIF1-α target and its inhibition by PDK originates pPDH, which can divert pyruvate to aerobic glycolysis instead of OXPHOS.^29^ In our study, pPDH was expressed by most OPMD cases, but had low values in OSCC samples. When conducting an analysis of OSCC and OSCC precursor lesions (simple hyperplasia and squamous intraepithelial neoplasia), Grimm, et al.^30^ (2016) reported the same association regarding PDH, but not pPDH. Studies on PDH expression in OSCC and other malignancies are scarce, although a study has been conducted on the pyruvate dehydrogenase complex for therapeutic purposes.^31^ Interestingly, when considering the hypoxic region of OSCC sections in our study, pPDH expression was lower in larger tumors and nearly associated with a higher DFS rate. This is intriguing and deserves further investigation.

LDHA, by preferentially converting pyruvate to lactate, is frequently overexpressed in cancer and associated with aggressive features and poor prognosis,^32^ which was also observed in OSCC.^33^ In another study, LDHA increased expression clearly associated with the process of oral carcinogenesis.^24^ Our results are in line with these findings, since LDHA expression frequencies were similar in OPMD and OSCC sections. In the latter, LDHA positivity was associated with poor clinicopathological features. Several studies show that LDHA activity is increased in the serum of patients with leukoplakia and OC,^34,35^ as well as in the cancer sections of patients,^33^ and LDHA has been suggested as a salivary biomarker of OPMD^36^ and OSCC.^35^

MCT4 is a lactate transporter protein that helps cancer cells balance their internal acidosis. Studies evaluating MCT4 expression in OC found a significant association with large lesions and worse prognosis, suggesting it as a potential biomarker to predict the aggressiveness of OSCC and patient prognosis.^15^ The prognostic value of this biomarker has also been reported in other malignancies.^14^ In our study, no OPMD expressed MCT4, while 22.2% of OSCC lesions were positive for this biomarker. In opposition, Bisetto, et al.^37^ (2018) stated that MCT4 is essential in oral carcinogenesis, as it mediates progression from oral dysplasia to OSCC. Positive MCT4 expression in OSCC was mostly observed in our study in advanced-stage poorly differentiated tumors, which was more evident in the analysis of the hypoxic region. As aforementioned, this low-affinity transporter preferentially exports lactate from highly glycolytic cells. Along with MCT1, MCT4 is the most studied isoform of the *SLC16* gene family, a family of transporters that couples the bidirectional movement of monocarboxylates and downward H^+^ concentration gradients. MCT1 preferentially uptakes lactate to fuel oxidative phosphorylation.^38^ The occurrence of a metabolic symbiosis between oxidative MCT1+ and MCT4+ glycolytic cancer cells has been reported,^14^ including in HNC.^39^ It would be interesting to explore this concept in this study.

CAIX is a direct HIF-1α target that is induced by aerobic glycolysis in order to allow cancer cells to adapt to the acidic microenvironment. It is also known as an intrinsic marker of hypoxia.^40^ This protein associates with tumor progression, metastasis, and poor prognosis^41^ in OC patients. It has been shown that CAIX might act as a predictive marker of hypoxia for malignant conversion in OC.^40^ This biomarker also showed a diagnostic value in the study by Pérez-Sayáns, et al.^42^ (2015) due to its high specificity in identifying dysplastic lesions in premalignant conditions. This was not observed in our study. In fact, CAIX was clearly overexpressed in malignant tissues when compared with OPMD, and nearly predicted a worse outcome. However, it was not possible to distinguish CAIX expression between normoxic and hypoxic regions.

The integrative analysis of the immunolabelling results allowed us to observe expression patterns. A highly glycolytic phenotype, i.e., the concomitant positivity of four or more biomarkers, increasingly occurred with a simultaneous increase in the malignant potential of OPMD, from OLP to leukoplakia with high-grade dysplasia. Oral dysplasia carries a 12.1% risk of progression to cancer,^43^ which shows the importance of developing progression-predictive models and potential targets for early therapeutic intervention. The glycolysis-impairing drug metformin has been used preclinically and successfully to reduce the size and number of carcinogen-induced premalignant oral lesions^44^ and prevent oral carcinogenesis of OPMD in a phase II clinical trial [NCT02581137, reviewed in Deng, et al.^45^ (2022)]. Regarding OSCC cases, high GP acquisition was positively correlated with loss of differentiation (significant associations) and poor overall survival. This reinforces the occurrence of an activated Warburg phenotype in the microenvironment of aggressive OSCC, in which multiple glycolysis-related biomarkers are involved. Combined signatures are increasingly proposed as useful methods for clinical categorization and individualized therapy for cancer patients, and the relevance of glycolysis-related proteins is frequently reported.^46,47^ This highlights the need to further investigate the glycolytic phenotype in the context of oral malignancies.

It is notable that this study had some limitations: the small sample size, the uneven distribution of the different groups, the semi-quantitative method used to evaluate immunohistochemistry results, and the absence of normal oral tissue samples. The treatment modalities, i.e., the chemotherapy regimens that the patients underwent, were not known, but it would have been interesting to study possible correlations between metabolic reprogramming and chemotherapy resistance, like other studies.^14^ Further investigation with a large cohort is needed to confirm the role of HIF-1α-targeted metabolic biomarkers as indicators of the malignant transformation of oral cells and poor prognosis of OSCC patients. These studies will be essential to develop further research on targeting these proteins as potential therapeutic strategies for OSCC patients. Few studies with *in vitro* and *in vivo* models have been conducted in this context. For instance, the expression of GLUT1, CAIX, HK2, and LDHA was observed in OSCC cell lines under hypoxic conditions, similarly to what was found in clinical samples.^24^ Downregulation of PKM2 in OSCC cell lines decreased proliferation, invasion, and apoptosis induction.^28^ Genetic or pharmacological disruption of LDHA decreased proliferation, migration, invasion, and EMT of OSCC cell lines, and inhibited tumor growth *in vivo*.^48^ The results obtained from these studies highlight the important role of the glycolytic metabolism in OSCC; however, more research is needed to independently validate the prognostic potential of these glycolytic biomarkers and their clinical use as therapeutic targets.

In conclusion, this study showed that oral cancer cells overexpress glycolysis-related proteins and this associates with aggressive features, which supports a hyperglycolytic phenotype in this type of cancer. We highlight the potential of HK2 as a prognostic biomarker. We also support that it might be useful to look for GLUT1 and GLUT3 immunoexpression in the hypoxic region of OSCC sections, as they seem to have prognostic value in this tumor section.
